# CELL5M: A geospatial database of agricultural indicators for Africa South of the Sahara

**DOI:** 10.12688/f1000research.9682.1

**Published:** 2016-10-10

**Authors:** Jawoo Koo, Cindy M. Cox, Melanie Bacou, Carlo Azzarri, Zhe Guo, Ulrike Wood-Sichra, Queenie Gong, Liangzhi You

**Affiliations:** 1 Environment and Production Technology Division, International Food Policy Research Institute (IFPRI), Washington, D.C., 20006-1002, USA

**Keywords:** spatial database, Africa South of Sahara, agricultural development, geographical information systems, data analysis

## Abstract

Recent progress in large-scale georeferenced data collection is widening opportunities for combining multi-disciplinary datasets from biophysical to socioeconomic domains, advancing our analytical and modeling capacity. Granular spatial datasets provide critical information necessary for decision makers to identify target areas, assess baseline conditions, prioritize investment options, set goals and targets and monitor impacts. However, key challenges in reconciling data across themes, scales and borders restrict our capacity to produce global and regional maps and time series. This paper provides overview, structure and coverage of CELL5M—an open-access database of geospatial indicators at 5 arc-minute grid resolution—and introduces a range of analytical applications and case-uses. CELL5M covers a wide set of agriculture-relevant domains for all countries in Africa South of the Sahara and supports our understanding of multi-dimensional spatial variability inherent in farming landscapes throughout the region.

## Highlights

- Spatial datasets for development are often disciplinary and not interoperable
- Developed CELL5M as a spatial database for agricultural research and development.
- Harmonized +750 multi-discipline data layers at 5 arc-minute resolution
- Key themes include food production, agroecology, demographics, and market access
- Since 2010, CELL5M has been used in more than 100 published studies

## Introduction

Over 70 percent of the population in Africa South of the Sahara (SSA) live in rural areas, their livelihood and food security often depending on smallholdings and rainfed agriculture ( Livingstone *et al.*, 2011). Many are also farming some of the most degraded soils in the world ( Cox & Koo, 2014), a challenge exacerbated by over-reliance on low-yielding crop varieties ( Mueller *et al.*, 2012) and inadequate market infrastructure ( Guo & Cox, 2014). Erratic shifts in weather and climate-related shocks are particularly hard felt in the region ( Challinor *et al.*, 2007). Development practitioners recognize that Africa’s economic development largely hinges on smallholder investment through improved agricultural yields, nutrition, ecosystem services and marketing opportunities ( Dixon *et al.*, 2001). Historically, however, there has been a lack of reliable, granular data to inform and monitor food and agricultural policies at appropriate scales. With the launch of the Sustainable Development Goals (SDGs) ( http://unstats.un.org/sdgs) —including zero global poverty and hunger by 2030—more granular, global and regional-level data need to reach decision makers for monitoring countries’ progress toward the goals.

Recent progress in georeferenced data collection and dissemination has widened access to multi-disciplinary datasets and created opportunities to advance data analytics ( Azzarri *et al.*, 2016). As data capacity improves, however, the potential of georeferenced socioeconomic datasets has not been fully utilized ( Azzarri *et al.*, 2016). A key challenge is reconciling and harmonizing multi-disciplinary indicators that can inform agricultural investments across scales and borders. To this end, HarvestChoice ( http://harvestchoice.org), a joint project between the International Food Policy Research Institute (IFPRI) and the University of Minnesota, developed the CELL5M database ( http://dx.doi.org/10.7910/DVN/G4TBLF), an open access catalog of georeferenced baseline indicators covering a broad range of agriculture-relevant domains. In this paper, we provide an overview of CELL5M and present a range of tools and applications for spatial targeting and strategic decision-making.

## CELL5M Overview

### What is CELL5M?

CELL5M is a geospatial database of biophysical and socioeconomic indicators for SSA covering four broad research domains: agriculture, agroecology, demographics and markets ( Table 1). All indicators are referenced to a uniform geographical information systems (GIS) grid: a flat table populated by over 300,000 grid cells overlaying SSA at 5 arc-minute spatial resolution. Each grid cell (or pixel) is approximately 10 kilometer × 10 kilometer and holds a stack of georeferenced data layers. CELL5M currently consists of over 750 data layers, providing a unique platform for multi-faceted analysis and fine-grain visualization at the nexus of agriculture and economic development. The database serves as the core to a decision-support system enabling development practitioners and analysts to explore complex relationships between major agroecological challenges ( *e.g*., soil and land degradation) and socioeconomic trends ( *e.g.*, poverty, health, and nutrition) ( Azzarri *et al.*, 2016). The structure of CELL5M allows for simplified numerical aggregations of gridded data along specific geographic domains, either sub-nationally ( *e.g*., across administrative boundaries, agroecological zones or watersheds) or across country borders for regional analyses ( *e.g.*, Omamo *et al.*, 2006)—all readily possible without GIS software. Users can visualize CELL5M indicators through HarvestChoice Mappr ( http://harvestchoice.org/mappr) or download from HarvestChoice Dataverse at http://dataverse.harvard.edu/dataverse/harvestchoice ( HarvestChoice, 2016a).

**Table 1.** CELL5M data layers by category, sub-category, and quantity thereof (as of April 2016).

Category	Sub-category (Number of data layers)
Agriculture	Harvested Area of Crops (134) Crop Production (134) Value of Crop Production (134) Crop Yield (134) Crop Yield Variability (2) Livestock (16)
Agroecology	Agroecological Zones (4) Climate (7) Elevation (1) Farming Systems (2) Land Cover and Land Use (21) Pests and Diseases (8) Soil Resources (19)
Demographics	Health and Nutrition (90) Income and Poverty (36) Population (12)
Markets	Marketshed (1) Portshed (1) Travel Time (11)

### Systematic assignment of grid cell ID

To refer to a cell’s boundary at any given spatial resolution, we created a universal identification system based on a basic unit of spatial analysis: the global grid cell ( HarvestChoice, 2016b). In GIS, one typically uses coordinates (latitude and longitude) of the upper-left and lower-right corners of the grid cell’s bounding box, or coordinates of the centroid, along with information on the projection system. To simplify identification, we universally label each cell as a sequential integer number, or grid cell ID. The grid cell ID can facilitate raster-based data analyses, aggregations and data sharing. The upper-left corner of the grid (longitude: -180.0, latitude: 90.0) starts at zero and ends at 9,331,199 in the lower-right corner (longitude: 180.0, latitude: -90.0). This system also allows for the grid cell ID to be mathematically computed at specific locations and converted to different resolutions and projection systems. This grid cell ID is used internally as the primary key of CELL5M database tables.

### Data harmonization and standardization

**Figure 1.**
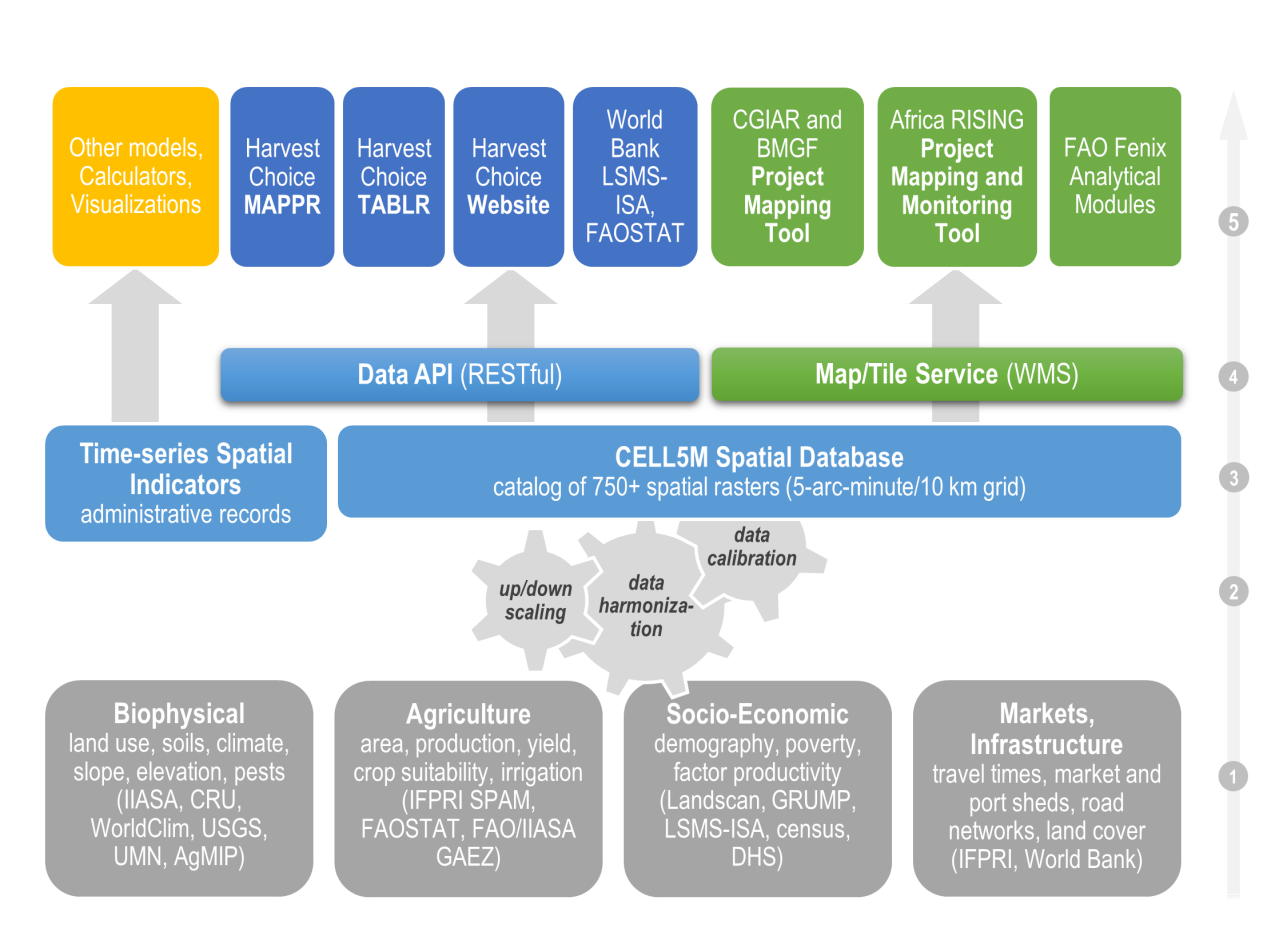
Schema of HarvestChoice open-data platform and CELL5M database. Using a variety of data sources and methods, CELL5M covers four broad research domains: biophysical, agricultural production, socio-economics and infrastructure (1). Using a combination of data resampling and harmonization routines (2), raw datasets are converted to a standard raster grid with a resulting set of uniform indicators across space and time (3). Indicators are distributed across platforms via application program interface and web mapping services (4). These services are freely and openly accessible through end-user tools ( *e.g*., Mappr and Tablr, available at http://harvestchoice.org/) and decision-support systems (5); Africa RISING, FAOSTAT, the World Bank’s Living Standards Measurement Study-Integrated Surveys on Agriculture (LSMS-ISA) and the Bill and Melinda Gates Foundation (BMGF) already consume CELL5M into their own analytical platforms.

CELL5M indicators originate from a variety of sources and partnerships, including CGIAR, World Bank, FAO, International Institute for Applied Systems Analysis (IIASA; http://iiasa.ac.at), Center for International Earth Science Information Network (CIESIN; http://ciesin.org), WorldClim ( http://worldclim.org), University of East Anglia ( http://cru.uea.ac.uk) and Africa Soil Information Service (AfSIS; http://africasoils.net). Raw datasets are provided in multiple spatio-temporal resolutions, geographical extents, and formats ( *e.g.*, tabular, vector and raster). They undergo harmonization routines that aim to generate standardized, cross-regional comparable statistics at uniform scale ( Figure 1). Raster and vector layers are typically re-projected to World Geodetic System (WGS) 84, a standard coordinate system for the Earth. Raster datasets of finer resolution ( *e.g*., 30 arc-second) are aggregated using weights ( *e.g*., land or population weights) or summarized ( *e.g*., population headcounts) to 5 arc-minute resolution. Conversely, we apply a disaggregation process when the source data is coarser, which is generally the case with socioeconomic datasets that are geo-referenced to administrative units. Where applicable, care is taken to ensure that country totals of disaggregated data are consistent with official national statistics. To maximize coverage across SSA, missing data are imputed using coarser statistics and prior information. The result is a stack of harmonized, interoperable datasets based on a standardized grid system. CELL5M complies with open-data standards ( Open Knowledge Foundation, 2016).

## Key data layers

This section provides additional methodological details on example key datasets included in CELL5M.

### Spatially-disaggregated crop production statistics

**Figure 2.**
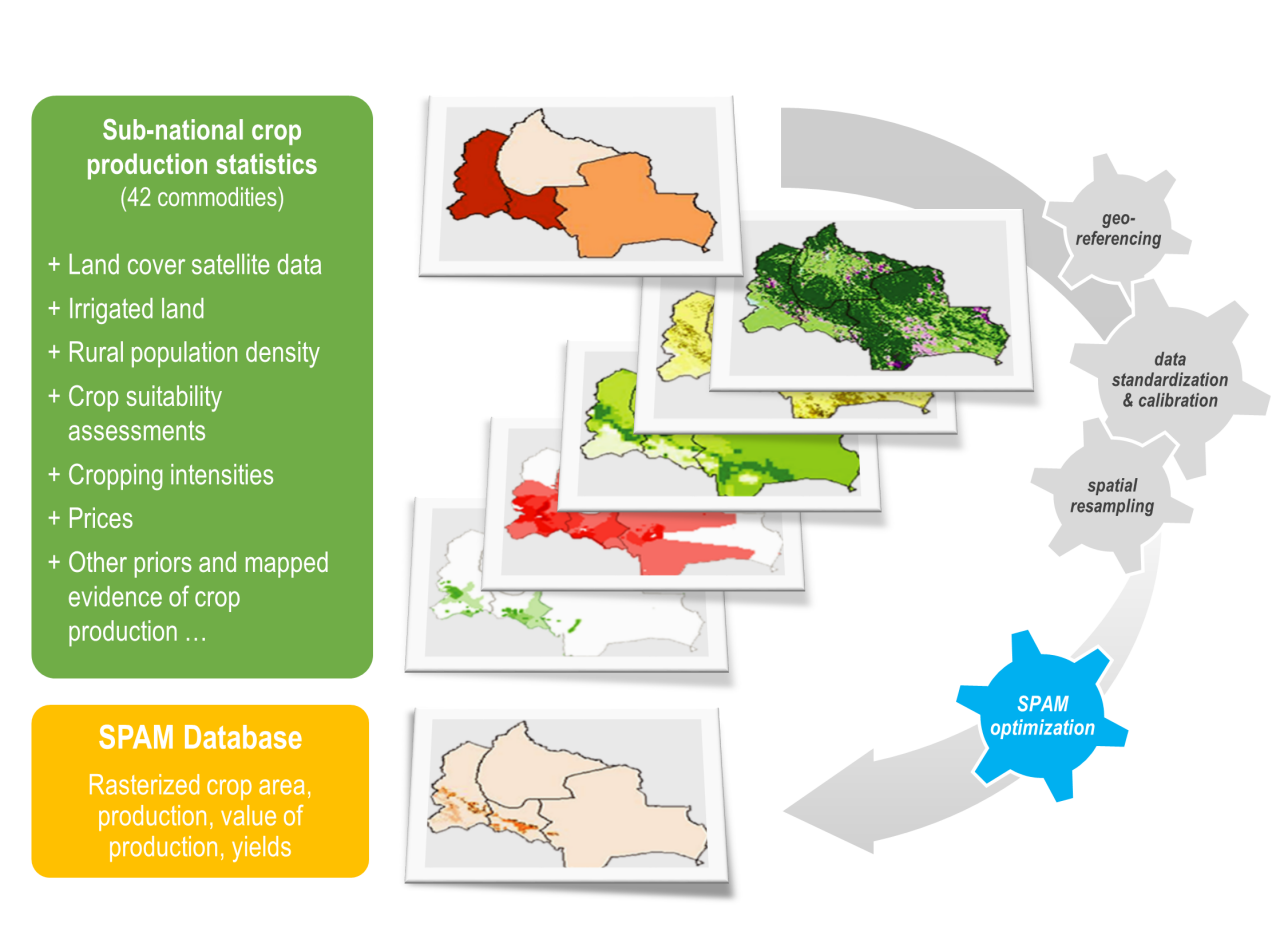
Mini-schema of the Spatial Production Allocation Model (SPAM). SPAM integrates information on crops ( *e.g*., subnational crop production statistics, land cover satellite-data, maps of irrigated areas, biophysical crop suitability assessments, population densities, cropping intensities and prices) and cross-entropy principles to allocate crops into individual pixels of a GIS database. The result for each pixel is the area (shown above), production, value of production and yield of each crop.

Beyond national-level assessments, spatially-disaggregated crop production statistics are the cornerstone of any analysis that explores the social, economic and environmental consequences of agricultural change and policies. The Spatial Production Allocation Model (SPAM) developed by the International food policy research institute (IFPRI) generates highly disaggregated, global distribution of area, production and yield for 42 commodities—accounting for 90 percent of the world’s crop production ( You *et al.*, 2014). To generate these data layers, geospatial information on crops—including subnational crop production statistics, satellite-derived land cover imagery, maps of irrigated areas, biophysical crop suitability assessments, population densities, cropping intensities and prices—is integrated to generate a set of prior estimates. These priors are then fed into an optimization model that applies cross-entropy principles, and area and production accounting constraints to allocate crops into individual pixels of a global grid at 5 arc-minute resolution ( You & Wood, 2006; You *et al.*, 2009) ( Figure 2). The result for each grid cell is the area, production, value of production, and yield of each crop, split by the shares grown under irrigated, high-input rainfed, low-input rainfed and subsistence rainfed conditions. CELL5M includes the SSA extent of SPAM; global coverage of SPAM data layers are available at http://mapspam.info.

### Market accessibility

**Figure 3.**
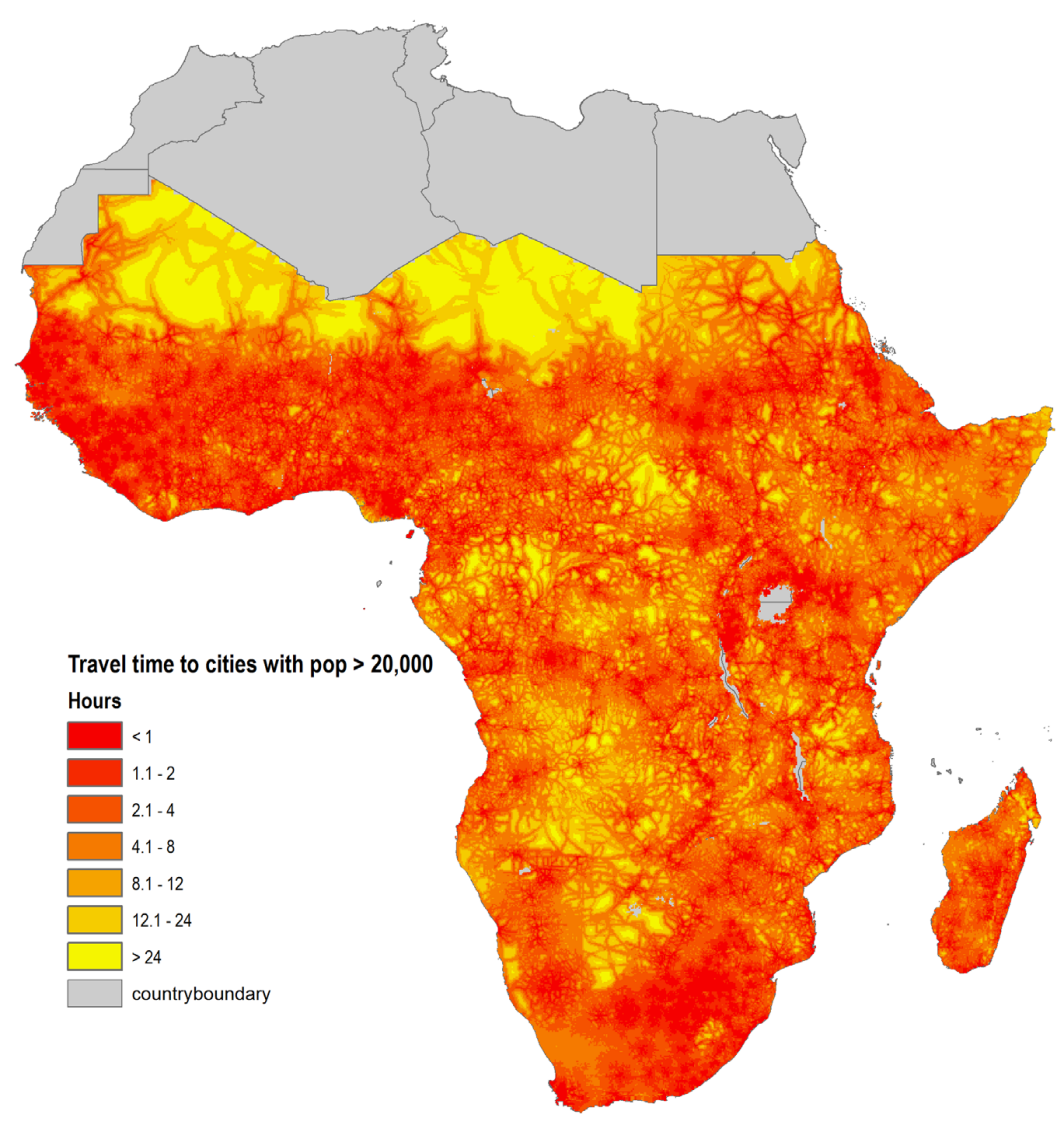
Market accessibility based on travel time to cities with populations greater than 20,000. We estimate travel time to nearest market centers (cities) of different population sizes using a spatial cost-distance algorithm and a combination of global spatial data layers including road network and type, elevation, slope, country boundaries, water and land cover. Source: Authors (available from CELL5M).

Farm households need access to markets to support agricultural and rural development, particularly in poorer regions. Challenging road conditions and inadequate infrastructure add to travel time and transportation cost, limiting farmers’ opportunity to purchase inputs and sell produce from remote crop production areas. The conventional method of measuring the Euclidean distance between two points in space ( *i.e*., farm-gate and market) ignores the terrain, road conditions and infrastructure status, hence does not accurately capture travel time. Estimates of the travel time to markets provide a better proxy for market accessibility since they combine distance with other information including road quality, slope, land cover, and mode of transportation ( Guo & Cox, 2014). To estimate market accessibility, we first identify the locations of different market centers and their sizes using population estimates from the Global Rural Urban Mapping Project ( CIESIN *et al.*, 2011). Then the travel times from farm-gate to the nearest cities of different population sizes are calculated using a spatial cost-distance algorithm and a combination of global spatial data layers including road network and type, elevation, slope, country boundaries, and land cover. CELL5M includes travel times to markets where populations are 20K ( Figure 3), 50K, 100K, 250K, and at least 500K.

### Subnational poverty

**Figure 4.**
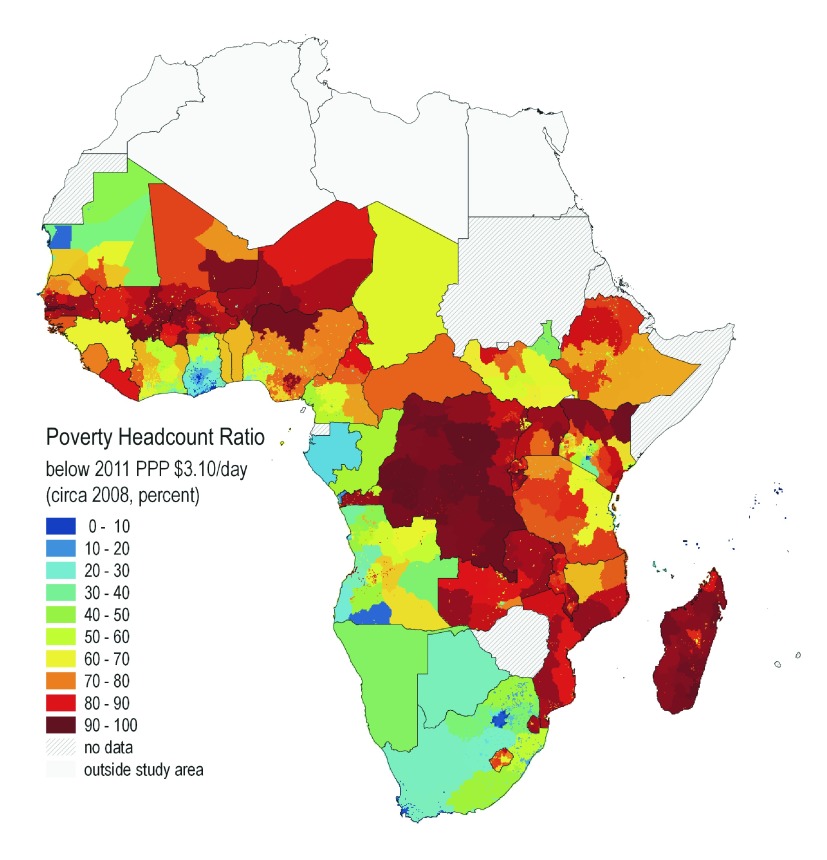
Poverty headcount ratios based on $3.10 poverty line. Ratios are derived from a series of 41 nationally representative household surveys conducted around 2008 for the majority of countries. Monthly per capita expenditure is converted to 2011 PPP dollars and a series of derived poverty statistics are estimated and mapped across all representative administrative units. Each survey map is rasterized to a uniform 5-arc-minute grid. Urban and rural estimates are applied to rural and urban grid cells, respectively. Source: Authors (available from CELL5M).

Poverty data layers in CELL5M are based on the comparison between household per-capita consumption expenditure and the $1.90 or $3.10/per-capita/day poverty lines ( Figure 4), expressed in international equivalent purchasing power parity (PPP) dollars, circa 2011 ( World Bank, 2014). By basing indicators on nationally- and regionally-representative household survey data, such as Household Income and Consumption Expenditure Survey (HICE), Integrated Household Survey (IHS), and Living Standards Measurement Study (LSMS), we avoid challenges with methods that combine national accounts and microdata ( Chen & Ravallion, 2008; Deaton, 2005; Ravallion, 2003). Using microdata with expansion factors and national PPP adjustments guarantees the validity of national and subnational estimates and, along with data harmonization, allows cross-country and time comparisons based on the purchasing power of the local currency in each survey year. Results are further validated by comparing the statistics calculated from microdata with official national indicators reported by World Bank’s PovcalNet ( http://iresearch.worldbank.org/PovcalNet). CELL5M includes 36 individual poverty and income data layers disaggregated across rural and urban domains.

### Simulated crop productivity changes

HarvestChoice’s grid-based crop modeling platform uses the Decision Support System for Agrotechnology Transfer (DSSAT) ( Hoogenboom *et al.*, 2009; Jones *et al.*, 2003) to simulate crop growth and yield. The platform integrates biophysical data layers from CELL5M ( *e.g*., crop geography, crop performance baseline, soil properties and climate characteristics) and estimates crop productivity response under various ‘what-if’ scenarios of change in agroecological conditions and farm management practices ( *e.g.*, maize profitability in response to doubling fertilizer application rates). The modeling platform has been used, for example, in ex-ante impact assessments of climate change ( Nelson *et al.*, 2009), agricultural technologies ( Rosegrant *et al.*, 2014), and climate variability associated with regional drought ( Cervigni & Morris, 2016). CELL5M includes model-derived indicators on maize yield variability in low and high-input rainfed production systems ( Koo & Cox, 2014).

## CELL5M use-cases

Well over 100 published manuscripts have used CELL5M datasets since 2010, from various institutions around the globe ( *e.g*., see Table 2). For example, CELL5M has been utilized to define and characterize study areas ( *e.g*., van Wart *et al.*, 2013); estimate market travel times ( *e.g.*, Damania *et al.*, 2016); explore geography changes in crop production ( *e.g*., Beddow & Pardey, 2015); calculate local agricultural commodity prices ( *e.g.*, Fjelde, 2015); map the threat of potential plant diseases ( *e.g*., Kriticos *et al.*, 2015); model climate change adaptations in agriculture ( *e.g*., Robinson *et al.*, 2015); and as a general data framework ( *e.g.*, Kwon *et al.*, 2016). CELL5M datasets have also been widely used in GIS training courses at academic institutions ( *e.g*., Deshazor, 2014), research grant proposals ( *e.g*., Ousmane Badiane, personal communication, January 28, 2016), and agricultural development investment strategies ( *e.g*., Stanley R. Wood, personal communication, February 12, 2016). The following sections describe examples in which partner organizations consume CELL5M to support food policy-relevant analyses.

**Table 2.** Selected publications (from 2010 through August 2016) that used CELL5M for underlying data. Bibliography of the publications can be found in the Supplementary Information.

Category	Number of Publications
Agriculture	71
Agroecology	41
Demographics	10
Markets	13
Boundaries	3

### Agricultural development domains

The Association for Strengthening Agricultural Research in Eastern and Central Africa (ASARECA) overlaid three key geospatial data layers from CELL5M—population density, market accessibility and agricultural potential—to construct ‘Agricultural Development Domains’ for investment targeting. Each domain is a distinct geographic area, where agricultural conditions ( *e.g.*, demographics, infrastructure and agroecology) are relatively homogeneous and distinguishable from others. CELL5M helps ASARECA to prescribe domain-specific interventions and evaluate their impacts accordingly ( Johnson & Flaherty, 2010). While grid cell-level information is necessary to generate flexible aggregations across space, CELL5M is particularly useful for such analysis because of the mixed nature of the datasets. This domain approach guides ASARECA’s upscaling of agricultural technologies across their target region in East and Central African countries ( Omamo *et al.*, 2006). For example, a spatially-explicit understanding of market accessibility is underlying the development of interventions linking value-chain actors with producers. A similar domain-based approach was used to analyze the biophysical suitability of agricultural innovations to local contexts ( *e.g*., Cox *et al.*, 2015).

### Agriculture and nutrition outcomes

**Figure 5.**
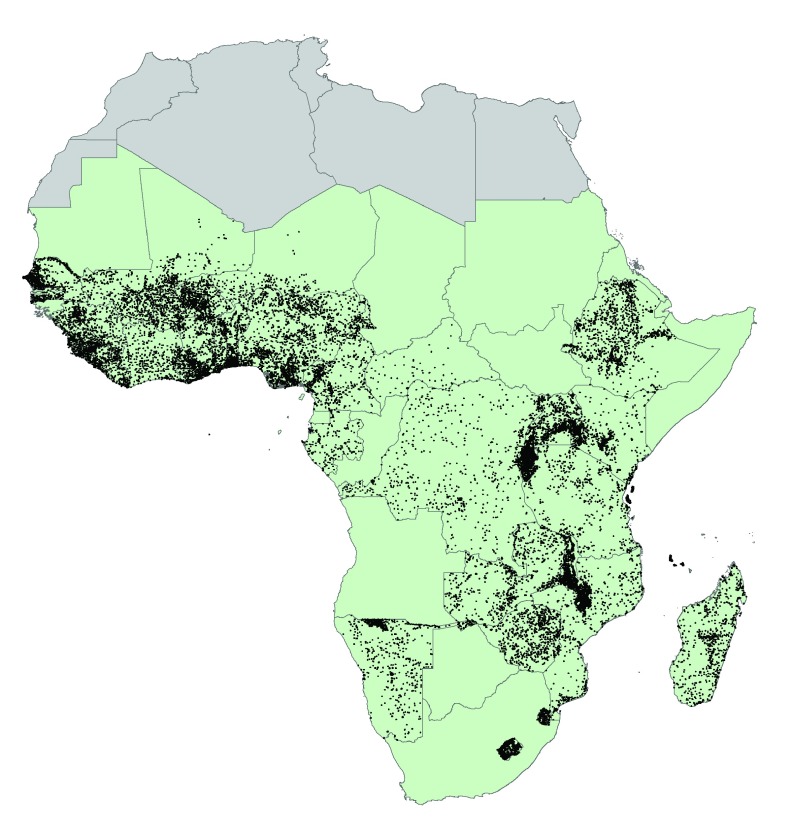
Cluster locations of Demographic Health Surveys (DHS) in Sub-Saharan Africa. There are 28,866 clusters across 32 countries. IFPRI’s AReNA (Advancing Research on Nutrition and Agriculture) initiative used datasets extracted from CELL5M for each cluster location in a series of econometric analyses to investigate the relationship between agriculture and nutrition outcomes. Source: Authors.

The last decade has witnessed a surge of interest in leveraging agricultural development for better nutrition. However, there is a dearth of rigorous evidence and policy-relevant research on agriculture-nutrition linkages ( Pinstrup-Andersen, 2013). As part of the Advancing Research on Nutrition and Agriculture (AReNA) initiative, HarvestChoice overlaid CELL5M indicators to an extensive series of georeferenced Demographic and Health Surveys (DHS; http://www.dhsprogram.com). Figure 5 shows the location of 28,866 clusters in SSA. Combining such datasets allows for more advanced econometric analyses to explore, for example, the spatial relationships between farming systems, biophysical characteristics, agricultural performance, market access and rural diets. For example, by overlaying agroecological indicators from CELL5M with childhood stunting data from DHS, Azzarri *et al*. (2016) showed that early childhood wasting is significantly more prevalent in the arid and semi-arid zones of SSA.

### Typology of food production systems

**Figure 6.**
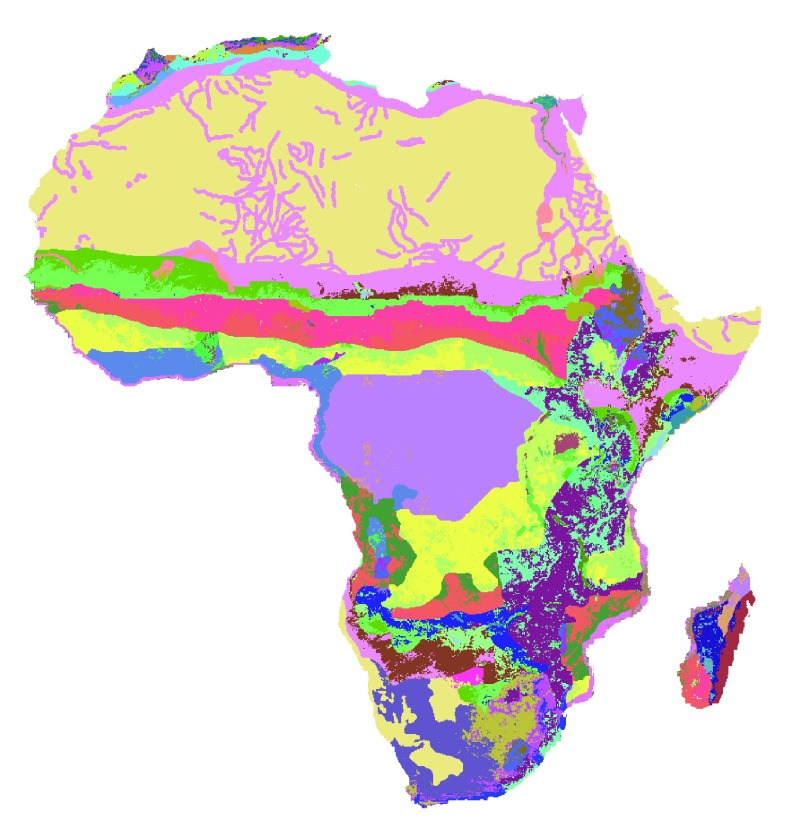
Distribution of Agricultural Production Zones (APZs) throughout Africa. Compared to maps of farming systems ( Dixon *et al.*, 2001). APZ provides a finer distinction across the continent by further disaggregating farming systems according to the data retrieved from CELL5M and the intensity of vegetation and non-vegetation observed from satellite-based remote sensing data. The map highlights considerable variations of biophysical conditions within countries and agroecological zones, representing over 300 different classifications of APZs (see Guo & Yu, 2015, for more details on the legend).

Africa has a rich landscape of farming systems and agricultural biodiversity. This diversity presents a challenge for quantitative analyses at regional scale. In Benin *et al*. (2011), data layers from CELL5M were used to construct a typology of food production systems across SSA. Agricultural productivity zones (APZs) were developed by first intersecting farming systems ( Dixon *et al.*, 2001) with other indicators related to natural endowment and socioeconomic development, calculated from data retrieved from CELL5M and then applying spatial clustering techniques ( Guo & Yu, 2015). The resulting APZs ( Figure 6) provide a more refined set of spatially-explicit typologies, compared to conventional country-level typologies, and allow policy makers to refine agricultural investment strategies.

### Tools for visualization and spatial analyses

CELL5M serves as the core database powering a growing number of open-access tools (see the list at http://harvestchoice.org/products/tool) and third-party applications reaching out to multiple audiences from research analysts to decision makers ( Figure 1). Gridded datasets are particularly easy to store in numerical matrices making them relatively manageable and simple to query. This allows us to serve CELL5M indicators through a RESTful Application Programming Interface (API), which allows computer programs to access and query CELL5M data using HTTP requests. CELL5M’s centroid coordinates ( *i.e*., latitude and longitude) may be used to graph and summarize indicators using simple visualization tools ( *e.g*., Tableau® or Microsoft Excel). Web-based interactive tools developed by HarvestChoice, for example Mappr ( http://harvestchoice.org/mappr) and Tablr ( http://harvestchoice.org/tablr) use the API to return tabular, graphical and spatial representations of CELL5M indicators. CELL5M raster layers are also served through a series of map services and may be queried via any GIS software compatible with OGC Web Map Service Standard ( Open Geospatial Consortium, 2016) ( *e.g*., ArcMap, QGIS, Leaflet or GDAL). For GIS users, the gridded data is also available in common raster formats (GeoTIFF and Esri ASCII). The World Bank’s micro-level datasets from the Living Standards Measurement Study-Integrated Surveys on Agriculture (LSMS-ISA) program uses CELL5M services to retrieved data for each survey site, including agroecological and market accessibility characteristics, to enrich its own data products (communications with the LSMS-ISA team, March 19, 2015).

## Conclusions

Through open and transparent sharing of high-resolution, harmonized multi-disciplinary datasets, CELL5M supports our understanding of multi-dimensional spatial variability in farming landscapes throughout SSA and helps better target potential interventions. A growing list of use-cases shows that CELL5M’s reach has moved well beyond its initial scope and is now used by a larger pool of scientists and decision makers. With the double challenge of climate change mitigation and global food security, we anticipate an ever-growing demand for easy-to-access and easy-to-use, harmonized open datasets for agricultural research and economic development.

It is worth noting that many methodological shortcomings in harmonizing and imputing raw data from various sources still prevail. More research is required to develop reliable statistical methods to interpolate point-and administrative-level data and especially to generate reliable confidence intervals. This will also require more open datasets becoming available. Many institutions are already committed to freely open their agriculture and nutrition datasets, yet a broad community-wide effort is still needed to improve data interoperability and utilization ( GODAN, 2015).

With advances in earth monitoring systems and image frequency and resolution, data products such as CELL5M necessitate further, continued investments to ensure that new data sources are incorporated, updated, modeled, and thoroughly validated. In that context, increased engagement with the broader community of data scientists and users is necessary for future success. We anticipate further collaboration with other emerging global data initiatives and partnerships ( *e.g*., Global Partnership for Sustainable Development Data), especially those aimed at monitoring mechanisms towards achieving global development goals.

## Data availability

The data referenced by this article are under copyright with the following copyright statement: Copyright: © 2016 Koo J et al.

Data tables in CSV format, grouped by theme in 18 zip-archived files, are available to download from the IFPRI HarvestChoice Dataverse at http://dx.doi.org/10.7910/DVN/G4TBLF ( HarvestChoice, 2016a). Any analysis software capable of reading comma-separated values (CSV) files, such as Microsoft Excel or WMS-enabled GIS desktop tool (e.g. QGIS, ArcMap, matlab, Python, R, GDAL) can be used to analyze the data.

## Supplementary material

**Supplementary Information for Table 2.**

Selected publications (from 2010 through August 2016) that used CELL5M for underlying data.
