# Supplementary material for: CELL5M: A geospatial database of agricultural indicators for Africa South of the Sahara
Source: F1000Res. 2016 Oct 10;5:2490. [Version 1] doi: 10.12688/f1000research.9682.1 (PMC5105882; doi:10.12688/f1000research.9682.1)
Supplement: Supplementary file 1 [file f1000research-5-10433-s0000.tgz › aa980a86-9902-4ab0-bfd5-c6e3e303d17b.docx]

**Supplementary Information for Table 2**

Selected publications (from 2010 through August 2016) that used CELL5M for underlying data.

| **Category** | **Number of Publications** | **Citations** |
| --- | --- | --- |
| Agriculture | 71 | Adhikari et al., 2015; Ahmed et al., 2016; Albanito et al., 2014; Anderson et al., 2015, 2014; Ayling et al., 2012; Beddow et al., 2015; Beddow and Pardey, 2015; Blaes et al., 2016; Boer et al., 2012; Cox et al., 2015; D’Aoust et al., 2016; Damania et al., 2016; De Groote et al., 2016; Di and Yang, 2015; Dorosh et al., 2012; Duan et al., 2016, 2015; Eriyagama et al., 2014; Fand et al., 2015, 2014; Fjelde, 2015; Folberth et al., 2012; Franch et al., 2015; Fritz et al., 2015; Fujimori et al., 2014; Glotter et al., 2015; Hannah et al., 2013; Hasegawa et al., 2014; Hendriks et al., 2016; Herrera Campo et al., 2011; Herrero et al., 2013; Homann-Kee Tui et al., 2013; Johnson et al., 2014; Katic and Morris, 2016; Kleinwechter et al., 2016; Kostandini et al., 2016; Kriticos et al., 2015; Liu et al., 2010, 2013; Marwick et al., 2014; Min et al., 2014; Morell et al., 2016; Müller and Robertson, 2014; Mutiga et al., 2015; Nelson, 2010; Nelson et al., 2010; Phalan et al., 2013; Pingali et al., 2014; Robertson et al., 2013; Rosegrant et al., 2014; Rovere et al., 2014; Schmidt et al., 2010; Schmitz et al., 2014; See et al., 2015; Stanton et al., 2013; Stuch et al., 2013; Tan et al., 2014; Thornton et al., 2014; Thornton and Herrero, 2015; Valin, H., Frank, S., Pirker, J., Mosnier, A., Forsell, N., Havlik, 2014; van Bussel et al., 2015; Van Oort et al., 2014; van Wart et al., 2013; Vicente-Serrano et al., 2012; Wiebelt et al., 2013; Wu et al., 2011; Xiong et al., 2014; You et al., 2014a, 2014b, 2011 |
| Agroecology | 41 | Aljaryian and Kumar, 2016; Ayling et al., 2012; Benin, 2016; Ben-Yakir et al., 2013; Eriyagama et al., 2014; Estes et al., 2016; Folberth et al., 2013; Fullmer et al., 2014; Gulma, 2013; Hess et al., 2016; Hochrainer-Stigler et al., 2014; Husmann et al., 2015; Imran et al., 2013; Katic and Morris, 2016; Khadioli et al., 2014; Kleinwechter et al., 2016; Kornher and Kalkuhl, 2016; Kriticos et al., 2015; Kumar et al., 2016; Kwon et al., 2016a; MacCarthy et al., 2015; Mailafiya, 2015; Mutiga et al., 2015; Pyhajarvi et al., 2013; Rigolot et al., 2016; Rimhanen and Kahiluoto, 2014; Riskin et al., 2013; Robinson et al., 2015; Romero et al., 2012; Rupf et al., 2015; Torres-Pacheco et al., 2013; Tsujimoto et al., 2014; Tully et al., 2015; van der Velde et al., 2014; van Wart et al., 2013; Vann et al., 2015; Waha et al., 2013; Walter and Karssen, 2015; Wu et al., 2011; Xie et al., 2012; Yu et al., 2012 |
| Demographics | 10 | Azzarri et al., 2016; Desiere et al., 2015; Dube et al., 2013; Graw and Ladenburger, 2012; Imran et al., 2015, 2014; Johnson and Flaherty, 2010; Katic and Morris, 2016; Pingali et al., 2014; Schmidt et al., 2010 |
| Markets | 13 | Desiere et al., 2015; Harris and Orr, 2014; Husmann et al., 2015; Johnson and Flaherty, 2010; Katic and Morris, 2016; Nijbroek and Andelman, 2015; Omamo et al., 2006; Schmidt et al., 2010; Smale et al., 2016; Takeshima et al., 2013; Takeshima and Liverpool-Tasie, 2015; Tesfaye et al., 2015; Yu and Guo, 2015 |
| Boundaries | 3 | Houssou et al., 2016; Kwon et al., 2016b; Salmon et al., 2015 |

# **References**

Adhikari, U., Nejadhashemi, A.P., Woznicki, S.A., 2015. Climate change and eastern Africa: A review of impact on major crops. Food Energy Secur. 4, 110–132. doi:10.1002/fes3.61

Ahmed, K.F., Wang, G., You, L., Yu, M., 2016. Potential impact of climate and socioeconomic changes on future agricultural land use in West Africa. Earth Syst. Dyn. 7, 151–165. doi:10.5194/esdd-6-1129-2015

Albanito, F., Beringer, T., Corstanje, R., Poulter, B., Stephenson, A., Zawadzka, J., Smith, P., 2014. Carbon implications of converting cropland to bioenergy crops or forest for climate mitigation: A global assessment. GCB Bioenergy 8, 81–95. doi:10.1111/gcbb.12242

Aljaryian, R., Kumar, L., 2016. Changing global risk of invading greenbug Schizaphis graminum under climate change. Crop Prot. 88, 137–148. doi:10.1016/j.cropro.2016.06.008

Anderson, W., You, L., Wood, S., Wood-Sichra, U., Wu, W., 2015. An analysis of methodological and spatial differences in global cropping systems models and maps. Glob. Ecol. Biogeogr. 24, 180–191. doi:10.1111/geb.12243

Anderson, W., You, L., Wood, S., Wood-Sichra, U., Wu, W., 2014. A comparative analysis of global cropping systems models and maps, IFPRI Discussion Paper 1327. Washington, D.C. doi:10.2139/ssrn.2405699

Ayling, S., Ferguson, M., Rounsley, S., Kulakow, P., 2012. Information resources for cassava research and breeding. Trop. Plant Biol. 5, 140–151. doi:10.1007/s12042-012-9093-x

Azzarri, C., Bacou, M., Cox, C.M., Guo, Z., Koo, J., 2016. Subnational socio-economic dataset availability. Nat. Clim. Chang. 6, 115–116. doi:10.1038/nclimate2842

Beddow, J.M., Pardey, P.G., 2015. Moving matters: The effect of location on crop production, The Journal of Economic History, 75, 219-249. doi:10.1017/S002205071500008X

Beddow, J.M., Pardey, P.G., Chai, Y., Hurley, T.M., Kriticos, D.J., Braun, H.-J., Park, R.F., Cuddy, W.S., Yonow, T., 2015. Research investment implications of shifts in the global geography of wheat stripe rust. Nat. Plants 1, 15132. doi:10.1038/nplants.2015.132

Benin, S., 2016. Returns to agricultural public spending in Ghana: Cocoa versus noncocoa subsector. SSRN Electron. J. doi:10.2139/ssrn.2740508

Ben-Yakir, D., Chen, M., Sinev, S., Seplyarsky, V., 2013. Chilo partellus (Swinhoe) (Lepidoptera: Pyralidae) a new invasive species in Israel. J. Appl. Entomol. 137, 398–400. doi:10.1111/j.1439-0418.2012.01740.x

Blaes, X., Chomé, G., Lambert, M.-J., Traoré, P., Schut, A., Defourny, P., 2016. Quantifying fertilizer application response variability with VHR satellite NDVI time series in a rainfed smallholder cropping system of Mali. Remote Sens. 8, 531. doi:10.3390/rs8060531

Boer, I.J.M., Hoving, I.E., Vellinga, T. V, Ven, G.W.J., Leffelaar, P.A., Gerber, P.J., 2012. Assessing environmental impacts associated with freshwater consumption along the life cycle of animal products: The case of Dutch milk production in Noord-Brabant. Int. J. Life Cycle Assess. 18, 193–203. doi:10.1007/s11367-012-0446-3

Cox, C.M., Kwon, H.Y., Koo, J., 2015. The Biophysical Potential for Urea Deep Placement Technology in Lowland Rice Production Systems of Ghana and Senegal. IFPRI Discuss. Pap. 1448, IFPRI Discussion Paper 1448.

D’Aoust, O., Sterck, O., Verwimp, P., 2016. Who benefited from Burundi’s demobilization program? World Bank Econ. Rev. lhw033. doi:10.1093/wber/lhw033

Damania, R., Berg, C., Russ, J., Federico Barra, A., Nash, J., Ali, R., 2016. Agricultural technology choice and transport. Am. J. Agric. Econ. doi:10.1093/ajae/aav073

De Groote, H., Oloo, F., Tongruksawattana, S., Das, B., 2016. Community-survey based assessment of the geographic distribution and impact of maize lethal necrosis (MLN) disease in Kenya. Crop Prot. 82, 30–35. doi:10.1016/j.cropro.2015.12.003

Desiere, S., Niragira, S., D’Haese, M., 2015. Cow or goat? Population pressure and livestock keeping in Burundi. Agrekon 54, 23–42. doi:10.1080/03031853.2015.1084941

Di, L., Yang, Z., 2015. Special section guest editorial: Remote sensing and sensor networks for promoting agro-geoinformatics. J. Appl. Remote Sens. 8. doi:10.1117/1.JRS.8.085101

Dorosh, P., Wang, H.G., You, L., Schmidt, E., 2012. Road connectivity, population, and crop production in Sub-Saharan Africa. Agric. Econ. 43, 89–103. doi:10.1111/j.1574-0862.2011.00567.x

Duan, P., Qin, L., Wang, Y., He, H., 2016. Spatial pattern characteristics of water footprint for maize production in Northeast China. J. Sci. Food Agric. 96, 561–568. doi:10.1002/jsfa.7124

Duan, P., Qin, L., Wang, Y., He, H., 2015. Spatiotemporal correlations between water footprint and agricultural inputs: A case study of maize production in northeast China. Water 7, 4026–4040. doi:10.3390/w7084026

Dube, S., Scholes, R.J., Nelson, G.C., Mason-D, D., Palazzo, A., Sikhalazo Dube, A., Sikhalazo Dube, C., 2013. South African food security and climate change: Agriculture futures. Open-Assessment E-Journal 7, 2013–35. doi:10.5018/economics-ejournal.ja.2013-35

Eriyagama, N., Chemin, Y., Alankara, R., 2014. A methodology for quantifying global consumptive water use of coffee for sustainable production under conditions of climate change. J. Water Clim. Chang. 5, 128. doi:10.2166/wcc.2013.035

Estes, L.D., Searchinger, T., Spiegel, M., Tian, D., Sichinga, S., Mwale, M., Kehoe, L., Kuemmerle, T., Berven, A., Chaney, N., Sheffield, J., Wood, E.F., Caylor, K.K., 2016. Reconciling agriculture, carbon and biodiversity in a savannah transformation frontier. Philos. Trans. R. Soc. B Biol. Sci. 371, 20150316. doi:10.1098/rstb.2015.0316

Fand, B.B., Sul, N.T., Bal, S.K., Minhas, P.S., 2015. Temperature impacts the development and survival of common cutworm (Spodoptera litura): Simulation and visualization of potential population growth in India under warmer temperatures through life cycle modelling and spatial mapping. PLoS One 10, e0124682. doi:10.1371/journal.pone.0124682

Fand, B.B., Tonnang, H.E.Z., Kumar, M., Bal, S.K., Singh, N.P., Rao, D.V.K.N., Kamble, A.L., Nangare, D.D., Minhas, P.S., 2014. Predicting the impact of climate change on regional and seasonal abundance of the mealybug Phenacoccus solenopsis Tinsley (Hemiptera: Pseudococcidae) using temperature-driven phenology model linked to GIS. Ecol. Modell. 288, 62–78. doi:10.1016/j.ecolmodel.2014.05.018

Fjelde, H., 2015. Farming or fighting? Agricultural price shocks and civil war in Africa. World Dev. 67, 525–534. doi:10.1016/j.worlddev.2014.10.032

Folberth, C., Gaiser, T., Abbaspour, K.C., Schulin, R., Yang, H., 2012. Regionalization of a large-scale crop growth model for sub-Saharan Africa: Model setup, evaluation, and estimation of maize yields. Agric. Ecosyst. Environ. 151, 21–33. doi:10.1016/j.agee.2012.01.026

Folberth, C., Yang, H., Gaiser, T., Abbaspour, K.C., Schulin, R., 2013. Modeling maize yield responses to improvement in nutrient, water and cultivar inputs in sub-Saharan Africa. Agric. Syst. 119, 22–34. doi:10.1016/j.agsy.2013.04.002

Franch, B., Vermote, E.F., Becker-Reshef, I., Claverie, M., Huang, J., Zhang, J., Justice, C., Sobrino, J.A., 2015. Improving the timeliness of winter wheat production forecast in the United States of America, Ukraine and China using MODIS data and NCAR Growing Degree Day information. Remote Sens. Environ. 161, 131–148. doi:10.1016/j.rse.2015.02.014

Fritz, S., See, L., Mccallum, I., You, L., Bun, A., Moltchanova, E., Duerauer, M., Albrecht, F., Schill, C., Perger, C., Havlik, P., Mosnier, A., Thornton, P., Wood-Sichra, U., Herrero, M., Becker-Reshef, I., Justice, C., Hansen, M., Gong, P., Abdel Aziz, S., Cipriani, A., Cumani, R., Cecchi, G., Conchedda, G., Ferreira, S., Gomez, A., Haffani, M., Kayitakire, F., Malanding, J., Mueller, R., Newby, T., Nonguierma, A., Olusegun, A., Ortner, S., Rajak, D.R., Rocha, J., Schepaschenko, D., Schepaschenko, M., Terekhov, A., Tiangwa, A., Vancutsem, C., Vintrou, E., Wenbin, W., van der Velde, M., Dunwoody, A., Kraxner, F., Obersteiner, M., 2015. Mapping global cropland and field size. Glob. Chang. Biol. 21, 1980–1992. doi:10.1111/gcb.12838

Fujimori, S., Hasegawa, T., Masui, T., Takahashi, K., 2014. Land use representation in a global CGE model for long-term simulation: CET vs. logit functions. Food Secur. 6, 685–699. doi:10.1007/s12571-014-0375-z

Fullmer, D., Chetty, V., Warnick, S., 2014. How good is bad weather? Proc. Am. Control Conf. 2711–2716. doi:10.1109/ACC.2014.6859469

Glotter, M.J., Moyer, E.J., Ruane, A.C., Elliott, J.W., 2015. Evaluating the sensitivity of agricultural model performance to different climate inputs. J. Appl. Meteorol. Climatol. 151113145618001. doi:10.1175/JAMC-D-15-0120.1

Graw, V., Ladenburger, C., 2012. Mapping Marginality Hotspots: Geographical Targeting for Poverty Reduction, Center for Development Research Working Paper Series. doi:10.2139/ssrn.2237970

Gulma, U.L., 2013. An analysis of temporal rainfall variability in Argungu area over the last half climatic year (1995-2012): Implication on rainfed crop production. Acad. J. Interdiscip. Stud. doi:10.5901/ajis.2013.v2n12p117

Hannah, L., Ikegami, M., Hole, D.G., Seo, C., Butchart, S.H.M., Peterson, A.T., Roehrdanz, P.R., 2013. Global climate change adaptation priorities for biodiversity and food security. PLoS One 8, e72590. doi:10.1371/journal.pone.0072590

Harris, D., Orr, A., 2014. Is rainfed agriculture really a pathway from poverty? Agric. Syst. 123, 84–96. doi:10.1016/j.agsy.2013.09.005

Hasegawa, T., Fujimori, S., Masui, T., Matsuoka, Y., 2014. Introducing detailed land-based mitigation measures into a computable general equilibrium model. J. Clean. Prod. 114, 233–242. doi:10.1016/j.jclepro.2015.03.093

Hendriks, C.M.J., Stoorvogel, J.J., Claessens, L., 2016. Exploring the challenges with soil data in regional land use analysis. Agric. Syst. 144, 9–21. doi:10.1016/j.agsy.2016.01.007

Herrera Campo, B.V., Hyman, G., Bellotti, A., 2011. Threats to cassava production: known and potential geographic distribution of four key biotic constraints. Food Secur. 3, 329–345. doi:10.1007/s12571-011-0141-4

Herrero, M., Havlík, P., Valin, H., Notenbaert, A., Rufino, M.C., Thornton, P.K., Blümmel, M., Weiss, F., Grace, D., Obersteiner, M., 2013. Biomass use, production, feed efficiencies, and greenhouse gas emissions from global livestock systems. Proc. Natl. Acad. Sci. 110, 20888–93. doi:10.1073/pnas.1308149110

Hess, T.M., Sumberg, J., Biggs, T., Georgescu, M., Haro-Monteagudo, D., Jewitt, G., Ozdogan, M., Marshall, M., Thenkabail, P., Daccache, A., Marin, F., Knox, J.W., 2016. A sweet deal? Sugarcane, water and agricultural transformation in Sub-Saharan Africa. Glob. Environ. Chang. 39, 181–194. doi:10.1016/j.gloenvcha.2016.05.003

Hochrainer-Stigler, S., van der Velde, M., Fritz, S., Pflug, G., 2014. Remote sensing data for managing climate risks: Index-based insurance and growth related applications for smallhold-farmers in Ethiopia. Clim. Risk Manag. 6, 27–38. doi:10.1016/j.crm.2014.09.002

Homann-Kee Tui, S., Blümmel, M., Valbuena, D., Chirima, A., Masikati, P., van Rooyen, A.F., Kassie, G.T., 2013. Assessing the potential of dual-purpose maize in southern Africa: A multi-level approach. F. Crop. Res. 153, 37–51. doi:10.1016/j.fcr.2013.07.002

Houssou, N., Johnson, M., Kolavalli, S., Asante-Addo, C., 2016. Changes in Ghanaian farming systems: Stagnation or a quiet transformation? SSRN Electron. J. doi:10.2139/ssrn.2740525

Husmann, C., Von Braun, J., Badiane, O., Akinbamijo, Y., Abiodun, F.O., Virchow, D., 2015. Tapping potentials of innovation for food security and sustainable agricultural growth: An Africa-wide perspective. SSRN Electron. J. 2701830. doi:10.2139/ssrn.2701830

Imran, M., Stein, A., Zurita-Milla, R., 2015. Using geographically weighted regression kriging for crop yield mapping in West Africa. Int. J. Geogr. Inf. Sci. 29, 234–257. doi:10.1080/13658816.2014.959522

Imran, M., Stein, A., Zurita-Milla, R., 2014. Investigating rural poverty and marginality in Burkina Faso using remote sensing-based products. Int. J. Appl. Earth Obs. Geoinf. 26, 322–334. doi:10.1016/j.jag.2013.08.012

Imran, M., Zurita-Milla, R., Stein, A., 2013. Modeling crop yield in West-African rainfed agriculture using global and local spatial regression. Agron. J. 105, 1177–1188. doi:10.2134/agronj2012.0370

Johnson, M., Benin, S., You, L., Diao, X., Chilonda, P., Kennedy, A., 2014. Exploring strategic priorities for regional agricultural research and development. IFPRI Discussion Paper 01318. Washington D.C. doi:10.2139/ssrn.2405713

Johnson, M., Flaherty, K., 2010. Strategic analysis and knowledge support systems for agriculture and rural development in Africa: Translating evidence into action. (Vol. 6) International Food Policy Research Institute (IFPRI), Washington, D.C.

Katic, P., Morris, J., 2016. Targeting investments in small-scale groundwater irrigation using Bayesian networks for a data-scarce river basin in Sub-Saharan Africa. Environ. Model. Softw. 82, 44–72. doi:10.1016/j.envsoft.2016.04.004

Khadioli, N., Tonnang, Z.E.H., Muchugu, E., Ong’amo, G., Achia, T., Kipchirchir, I., Kroschel, J., Le Ru, B., 2014. Effect of temperature on the phenology of Chilo partellus (Swinhoe) (Lepidoptera, Crambidae); simulation and visualization of the potential future distribution of C. partellus in Africa under warmer temperatures through the development of life-table param. Bull. Entomol. Res. 104, 809–822. doi:10.1017/S0007485314000601

Kleinwechter, U., Gastelo, M., Ritchie, J., Nelson, G., Asseng, S., 2016. Simulating cultivar variations in potato yields for contrasting environments. Agric. Syst. 145, 51–63. doi:10.1016/j.agsy.2016.02.011

Kornher, L., Kalkuhl, M., 2016. The Costs and Benefits of Regional Cooperation on Grain Reserves: The Case of ECOWAS, in: Food Price Volatility and Its Implications for Food Security and Policy. Springer International Publishing, Cham, pp. 353–384. doi:10.1007/978-3-319-28201-5_15

Kostandini, G., La Rovere, R., Guo, Z., 2016. Ex Ante Welfare Analysis of Technological Change: The Case of Nitrogen Efficient Maize for African Soils. Can. J. Agric. Econ. 64, 147–168. doi:10.1111/cjag.12067

Kriticos, D.J., Ota, N., Hutchison, W.D., Beddow, J., Walsh, T., Tay, W.T., Borchert, D.M., Paula-Moreas, S. V., Czepak, C., Zalucki, M.P., 2015. The potential distribution of invading Helicoverpa armigera in North America: Is it just a matter of time? PLoS One 10, e0119618. doi:10.1371/journal.pone.0119618

Kumar, S., Ramilan, T., Ramarao, C.A., Rao, C.S., Whitbread, A., 2016. Farm level rainwater harvesting across different agro climatic regions of India: Assessing performance and its determinants. Agric. Water Manag. 176, 55–66. doi:10.1016/j.agwat.2016.05.013

Kwon, H.-Y., Nkonya, E., Johnson, T., Graw, V., Kato, E., Kihiu, E., 2016a. Global Estimates of the Impacts of Grassland Degradation on Livestock Productivity from 2001 to 2011, in: Economics of Land Degradation and Improvement – A Global Assessment for Sustainable Development. Springer International Publishing, pp. 197–214. doi:10.1007/978-3-319-19168-3_8

Kwon, H.-Y., Nkonya, E., Johnson, T., Graw, V., Kato, E., Kihiu, E., 2016b. Global Estimates of the Impacts of Grassland Degradation on Livestock Productivity from 2001 to 2011, in: Economics of Land Degradation and Improvement – A Global Assessment for Sustainable Development. Springer International Publishing, pp. 197–214. doi:10.1007/978-3-319-19168-3_8

Liu, J., You, L., Amini, M., Obersteiner, M., Herrero, M., Zehnder, A.J.B., Yang, H., 2010. A high-resolution assessment on global nitrogen flows in cropland. Proc. Natl. Acad. Sci. 107, 8035–8040. doi:10.1073/pnas.0913658107

Liu, Z., Li, Z., Tang, P., Li, Z., Wu, W., Yang, P., You, L., Tang, H., 2013. Change analysis of rice area and production in China during the past three decades. J. Geogr. Sci. 23, 1005–1018. doi:10.1007/s11442-013-1059-x

MacCarthy, D.S., Akponikpe, P.B.I., Narh, S., Tegbe, R., 2015. Modeling the effect of seasonal climate variability on the efficiency of mineral fertilization on maize in the coastal savannah of Ghana. Nutr. Cycl. Agroecosystems 102, 45–64. doi:10.1007/s10705-015-9701-x

Mailafiya, D.M., 2015. Agrobiodiversity for Biological Pest Control in Sub-Saharan Africa, in: Sustainable Agriculture Reviews. Springer International Publishing, pp. 107–143. doi:10.1007/978-3-319-21629-4_4

Marwick, T.R., Borges, A.V., Van Acker, K., Darchambeau, F., Bouillon, S., 2014. Disproportionate contribution of riparian inputs to organic carbon pools in freshwater systems. Ecosystems 17, 974–989. doi:10.1007/s10021-014-9772-6

Min, M., Zhao, W., Hu, T., Chen, J., Nie, X., 2014. Influential factors of spatial distribution of wheat yield in China during 1978 - 2007: A spatial econometric analysis. IEEE J. Sel. Top. Appl. Earth Obs. Remote Sens. 7, 4453–4460. doi:10.1109/JSTARS.2014.2325898

Morell, F.J., Yang, H.S., Cassman, K.G., Wart, J. Van, Elmore, R.W., Licht, M., Coulter, J.A., Ciampitti, I.A., Pittelkow, C.M., Brouder, S.M., Thomison, P., Lauer, J., Graham, C., Massey, R., Grassini, P., 2016. Can crop simulation models be used to predict local to regional maize yields and total production in the U.S. Corn Belt? F. Crop. Res. 192, 1–12. doi:10.1016/j.fcr.2016.04.004

Müller, C., Robertson, R.D., 2014. Projecting future crop productivity for global economic modeling. Agric. Econ. 45, 37–50. doi:10.1111/agec.12088

Mutiga, S.K., Hoffmann, V., Harvey, J., Milgroom, M.G., Nelson, R., 2015. Assessment of aflatoxin and fumonisin contamination of maize in western Kenya. Phytopathology 105, 1250–1261. doi:10.1094/PHYTO-10-14-0269-R

Nelson, G.C., 2010. The perfect storm. Significance 7, 13–16. doi:10.1111/j.1740-9713.2010.00404.x

Nelson, G.C., Rosegrant, M.W., Palazzo, A., Gray, I., Ingersoll, C., Robertson, R.D., Tokgoz, S., Zhu, T., Sulser, T.B., Ringler, C., Msangi, S., You, L., 2010. Food security, farming, and climate change to 2050: Scenarios, results, policy options, Research reports IFPRI. International Food Policy Research Institute, Washington, D.C. doi:10.2499/9780896291867

Nijbroek, R.P., Andelman, S.J., 2015. Regional suitability for agricultural intensification: A spatial analysis of the Southern Agricultural Growth Corridor of Tanzania. Int. J. Agric. Sustain. 5903, 1–17. doi:10.1080/14735903.2015.1071548

Omamo, S.W., Diao, X., Wood, S., Chamberlin, J., You, L., Benin, S., Wood-Sichra, U., Tatwangire, A., 2006. Strategic Priorities for Agricultural Development in Eastern and Central Africa. International Food Policy Research Institute, Washington, D.C. doi:10.2499/9780896291584RR150

Phalan, B., Bertzky, M., Butchart, S.H.M., Donald, P.F., Scharlemann, J.P.W., Stattersfield, A.J., Balmford, A., 2013. Crop expansion and conservation priorities in tropical countries. PLoS One 8, e51759. doi:10.1371/journal.pone.0051759

Pingali, P., Schneider, K., Zurek, M., 2014. Poverty, Agriculture and the Environment: The Case of Sub-Saharan Africa, in: Marginality: Addressing the Nexus of Poverty, Exclusion and Ecology. Springer Netherlands, Dordrecht, pp. 151–168. doi:10.1007/978-94-007-7061-4_10

Pyhajarvi, T., Hufford, M.B., Mezmouk, S., Ross-Ibarra, J., 2013. Complex patterns of local adaptation in Teosinte. Genome Biol. Evol. 5, 1594–1609. doi:10.1093/gbe/evt109

Rigolot, C., de Voil, P., Douxchamps, S., Prestwidge, D., Van Wijk, M., Thornton, P., Rodriguez, D., Henderson, B., Medina, D., Herrero, M., 2016. Interactions between intervention packages, climatic risk, climate change and food security in mixed crop–livestock systems in Burkina Faso. Agric. Syst. doi:10.1016/j.agsy.2015.12.017

Rimhanen, K., Kahiluoto, H., 2014. Management of harvested C in smallholder mixed farming in Ethiopia. Agric. Syst. 130, 13–22. doi:10.1016/j.agsy.2014.06.003

Riskin, S.H., Porder, S., Neill, C., Figueira, A.M. e. S., Tubbesing, C., Mahowald, N., 2013. The fate of phosphorus fertilizer in Amazon soya bean fields. Philos. Trans. R. Soc. B Biol. Sci. 368, 20120154–20120154. doi:10.1098/rstb.2012.0154

Robertson, R., Nelson, G., Thomas, T., Rosegrant, M., 2013. Incorporating process-based crop simulation models into global economic analyses. Am. J. Agric. Econ. 95, 228–235. doi:10.1093/ajae/aas034

Robinson, S., Mason-D ’croz, D., Islam, S., Cenacchi, N., Creamer, B., Gueneau, A., Hareau, G., Kleinwechter, U., Mottaleb, K., Nedumaran, S., Robertson, R., Rosegrant, M.W., Sika, G., Sulser, T.B., Wiebe, K., 2015. Climate change adaptation in agriculture: Ex ante analysis of promising and alternative crop technologies using DSSAT and IMPACT. IFPRI Discussion Paper 01469. Washington, D.C.

Romero, C.C., Hoogenboom, G., Baigorria, G.A., Koo, J., Gijsman, A.J., Wood, S., 2012. Reanalysis of a global soil database for crop and environmental modeling. Environ. Model. Softw. 35, 163–170. doi:10.1016/j.envsoft.2012.02.018

Rosegrant, M.W., Koo, J., Cenacchi, N., Ringler, C., Robertson, R.D., Fisher, M., Cox, C.M., Garrett, K.A., Perez, N.D., Sabbagh, P., 2014. Food security in a world of natural resource scarcity The role of agricultural technologies. International Food Policy Research Institute (IFPRI), Washington, D.C. doi:10.2499/9780896298477

Rovere, R. La, Abdoulaye, T., Kostandini, G., Guo, Z., Mwangi, W., MacRobert, J., Dixon, J., 2014. Economic, production, and poverty impacts of investing in maize tolerant to drought in Africa: An ex-ante assessment. J. Dev. Areas 48, 199–225. doi:10.1353/jda.2014.0016

Rupf, G. V., Bahri, P.A., de Boer, K., McHenry, M.P., 2015. Barriers and opportunities of biogas dissemination in Sub-Saharan Africa and lessons learned from Rwanda, Tanzania, China, India, and Nepal. Renew. Sustain. Energy Rev. 52, 468–476. doi:10.1016/j.rser.2015.07.107

Salmon, J.M., Friedl, M.A., Frolking, S., Wisser, D., Douglas, E.M., 2015. Global rain-fed, irrigated, and paddy croplands: A new high resolution map derived from remote sensing, crop inventories and climate data. Int. J. Appl. Earth Obs. Geoinf. 38, 321–334. doi:10.1016/j.jag.2015.01.014

Schmidt, E., Dorosh, P., Wang, H.-G., You, L., 2010. Crop production and road connectivity in sub-Saharan Africa: A spatial analysis, World Bank Policy Research Working Papers. doi:10.1596/1813-9450-5385

Schmitz, C., van Meijl, H., Kyle, P., Nelson, G.C., Fujimori, S., Gurgel, A., Havlik, P., Heyhoe, E., D’Croz, D.M., Popp, A., Sands, R., Tabeau, A., van der Mensbrugghe, D., von Lampe, M., Wise, M., Blanc, E., Hasegawa, T., Kavallari, A., Valin, H., 2014. Land-use change trajectories up to 2050: insights from a global agro-economic model comparison. Agric. Econ. 45, 69–84. doi:10.1111/agec.12090

See, L., Fritz, S., Perger, C., Schill, C., McCallum, I., Schepaschenko, D., Duerauer, M., Sturn, T., Karner, M., Kraxner, F., Obersteiner, M., 2015. Harnessing the power of volunteers, the internet and Google Earth to collect and validate global spatial information using Geo-Wiki. Technol. Forecast. Soc. Change 98, 324–335. doi:10.1016/j.techfore.2015.03.002

Smale, M., Diressie, M.T., Birol, E., 2016. Understanding the potential for adoption of high-iron varieties of pearl millet in Maharashtra, India: What explains their popularity? Food Secur. 8, 331–344. doi:10.1007/s12571-016-0559-9

Stanton, M.C., Molyneux, D.H., Kyelem, D., Bougma, R.W., Koudou, B.G., Kelly-Hope, L.A., 2013. Baseline drivers of lymphatic filariasis in Burkina Faso. Geospat. Health 8, 159. doi:10.4081/gh.2013.63

Stuch, B., Schaldach, R., Schüngel, J., 2013. A Model Based Method to Assess Climate Change Impacts on Rain-Fed Farming Systems: How to Analyze Crop-Yield Variability?, in: Knowledge Systems of Societies for Adaptation and Mitigation of Impacts of Climate Change. Springer Berlin Heidelberg, pp. 489–510. doi:10.1007/978-3-642-36143-2_29

Takeshima, H., Liverpool-Tasie, L.S.O., 2015. Fertilizer subsidies, political influence and local food prices in sub-Saharan Africa: Evidence from Nigeria. Food Policy 54, 11–24. doi:10.1016/j.foodpol.2015.04.003

Takeshima, H., Nin-Pratt, A., Diao, X., 2013. Mechanization and agricultural technology evolution, agricultural intensification in Sub-Saharan Africa: Typology of agricultural mechanization in Nigeria. Am. J. Agric. Econ. 95, 1230–1236. doi:10.1093/ajae/aat045

Tan, J., Yang, P., Liu, Z., Wu, W., Zhang, L., Li, Z.Z., You, L., Tang, H., Li, Z.Z., 2014. Spatio-temporal dynamics of maize cropping system in Northeast China between 1980 and 2010 by using spatial production allocation model. J. Geogr. Sci. 24, 397–410. doi:10.1007/s11442-014-1096-0

Tesfaye, K., Jaleta, M., Jena, P., Mutenje, M., 2015. Identifying potential recommendation domains for conservation agriculture in Ethiopia, Kenya, and Malawi. Environ. Manage. 55, 330–346. doi:10.1007/s00267-014-0386-8

Thornton, P.K., Ericksen, P.J., Herrero, M., Challinor, A.J., 2014. Climate variability and vulnerability to climate change: A review. Glob. Chang. Biol. doi:10.1111/gcb.12581

Thornton, P.K., Herrero, M., 2015. Adapting to climate change in the mixed crop and livestock farming systems in sub-Saharan Africa. Nat. Clim. Chang. 5, 830–836. doi:10.1038/nclimate2754

Torres-Pacheco, I., López-Arroyo, J.I., Aguirre-Gómez, J.A., Guevara-González, R.G., Yänez-López, R., Hernández-Zul, M.I., Quijano-Carranza, J.A., 2013. Potential distribution in Mexico of Diaphorina citri (Hemiptera : Psyllidae) vector of Huanglongbing pathogen. Florida Entomol. 96, 36–47. doi:10.1653/024.096.0105

Tsujimoto, Y., Muranaka, S., Saito, K., Asai, H., 2014. Limited Si-nutrient status of rice plants in relation to plant-available Si of soils, nitrogen fertilizer application, and rice-growing environments across Sub-Saharan Africa. F. Crop. Res. 155, 1–9. doi:10.1016/j.fcr.2013.10.003

Tully, K., Sullivan, C., Weil, R., Sanchez, P., 2015. The state of soil degradation in sub-Saharan Africa: Baselines, trajectories, and solutions. Sustain. doi:10.3390/su7066523

Valin, H., Frank, S., Pirker, J., Mosnier, A., Forsell, N., Havlik, P., 2014. Improvements to GLOBIOM for modelling of biofuels indirect land use change. IIASA.

van Bussel, L.G.J., Grassini, P., Van Wart, J., Wolf, J., Claessens, L., Yang, H., Boogaard, H., de Groot, H., Saito, K., Cassman, K.G., van Ittersum, M.K., 2015. From field to atlas: Upscaling of location-specific yield gap estimates. F. Crop. Res. 177, 98–108. doi:10.1016/j.fcr.2015.03.005

van der Velde, M., Folberth, C., Balkovič, J., Ciais, P., Fritz, S., Janssens, I.A., Obersteiner, M., See, L., Skalský, R., Xiong, W., Peñuelas, J., 2014. African crop yield reductions due to increasingly unbalanced Nitrogen and Phosphorus consumption. Glob. Chang. Biol. 20, 1278–1288. doi:10.1111/gcb.12481

Van Oort, P.A.J., Saito, K., Tanaka, A., Amovin-Assagba, E., Van Bussel, L.G.J., van Wart, J., de Groot, H., van Ittersum, M.K., Cassman, K.G., Wopereis, M.C.S., 2014. Assessment of rice self-sufficiency in 2025 in eight African countries. Glob. Food Sec. 5, 39–49. doi:10.1016/j.gfs.2015.01.002

van Wart, J., van Bussel, L.G.J., Wolf, J., Licker, R., Grassini, P., Nelson, A., Boogaard, H., Gerber, J., Mueller, N.D., Claessens, L., van Ittersum, M.K., Cassman, K.G., 2013. Use of agro-climatic zones to upscale simulated crop yield potential. F. Crop. Res. 143, 44–55. doi:10.1016/j.fcr.2012.11.023

Vann, L., Kono, T., Pyhäjärvi, T., Hufford, M.B., Ross-Ibarra, J., 2015. Natural variation in teosinte at the domestication locus teosinte branched1 (tb1). PeerJ 3, e900. doi:10.7717/peerj.900

Vicente-Serrano, S.M., Beguería, S., Lorenzo-Lacruz, J., Camarero, J.J., López-Moreno, J.I., Azorin-Molina, C., Revuelto, J., Morán-Tejeda, E., Sanchez-Lorenz, A., 2012. Performance of drought indices for ecological, agricultural, and hydrological applications. Earth Interact. 16, 1–27. doi:10.1175/2012EI000434.1

Waha, K., Müller, C., Bondeau, A., Dietrich, J.P., Kurukulasuriya, P., Heinke, J., Lotze-Campen, H., 2013. Adaptation to climate change through the choice of cropping system and sowing date in sub-Saharan Africa. Glob. Environ. Chang. 23, 130–143. doi:10.1016/j.gloenvcha.2012.11.001

Walter, N.T., Karssen, G., 2015. The potential distribution and risk assessment of Pratylenchus zeae on maize in Belgium and the Netherlands. Adv. Plants Agric. Res. 2, 62. doi:10.15406/apar.2015.02.00062

Wiebelt, M., Breisinger, C., Ecker, O., Al-Riffai, P., Robertson, R., Thiele, R., 2013. Compounding food and income insecurity in Yemen: Challenges from climate change. Food Policy 43, 77–89. doi:10.1016/j.foodpol.2013.08.009

Wu, W., Tang, H., Yang, P., You, L., Zhou, Q., Chen, Z., Shibasaki, R., 2011. Scenario-based assessment of future food security. J. Geogr. Sci. 21, 3–17. doi:10.1007/s11442-011-0825-x

Xie, H., Longuevergne, L., Ringler, C., Scanlon, B.R., 2012. Calibration and evaluation of a semi-distributed watershed model of Sub-Saharan Africa using GRACE data. Hydrol. Earth Syst. Sci. 16, 3083–3099. doi:10.5194/hess-16-3083-2012

Xiong, W., Balkovič, J., van der Velde, M., Zhang, X., Izaurralde, R.C., Skalský, R., Lin, E., Mueller, N., Obersteiner, M., 2014. A calibration procedure to improve global rice yield simulations with EPIC. Ecol. Modell. 273, 128–139. doi:10.1016/j.ecolmodel.2013.10.026

You, L., Ringler, C., Wood-Sichra, U., Robertson, R., Wood, S., Zhu, T., Nelson, G., Guo, Z., Sun, Y., 2011. What is the irrigation potential for Africa? A combined biophysical and socioeconomic approach. Food Policy 36, 770–782. doi:10.1016/j.foodpol.2011.09.001

You, L., Wood, S., Wood-Sichra, U., Wu, W., 2014a. Generating global crop distribution maps: From census to grid. Agric. Syst. 127, 53–60. doi:10.1016/j.agsy.2014.01.002

You, L., Xie, H., Wood-Sichra, U., Guo, Z., Wang, L., 2014b. Irrigation potential and investment return in Kenya. Food Policy 47, 34–45. doi:10.1016/j.foodpol.2014.04.006

Yu, B., Guo, Z., 2015. Measurement of agricultural productivity in Africa South of Sahara: A spatial typology application. IFPRI Discussion Paper 01410. doi:10.2139/ssrn.2564537

Yu, Q., Wu, W., Yang, P., Li, Z., Xiong, W., Tang, H., 2012. Proposing an interdisciplinary and cross-scale framework for global change and food security researches. Agric. Ecosyst. Environ. 156, 57–71. doi:10.1016/j.agee.2012.04.026
