# Supplementary material for: CELL5M: A geospatial database of agricultural indicators for Africa South of the Sahara
Source: F1000Res. 2016 Oct 10;5:2490. [Version 1] doi: 10.12688/f1000research.9682.1 (PMC5105882; doi:10.12688/f1000research.9682.1)
Supplement: Peer review history

# Peer review: Referee response for version 1

**DOI:** 10.5256/f1000research.10433.r17508

**Published:** 2016-11-10

**Authors:** Katharina Waha

**Affiliations:** 1 Commonwealth Scientific and Industrial Research Organization (CSIRO), St Lucia, Qld, Australia

I’m following the HarvestChoice webpage for a while now and used data provided a few years ago, so it’s good to see the publication of their spatial database.

 The title and abstract are appropriate for the content of the article. The basic methods for generating CELL5M are explained, however I would like to ask the authors to not only describe the sources of key layers, but all data sources they have used (maybe in the SI). This description should include at least the input data for each data set, the original resolution or spatial units, the base year(s) and a reference to a full documentation.

 This is important in my opinion because at the moment you *reconcile data sets only spatially* but they might diverge in their methods and assumptions which prevents particular applications. A simple example are the livestock densities you use from the Gridded Livestock of the World 2007 which are modelled based on (among other things) climate as in WorldClim and the climate variables you present in CELL5M that use CRU. What happens often as well when developing a global data set is that areas are masked out or typologies of certain areas are created with distinct thresholds and if this information is not available to a user he might falsely interpret a spatial overlay of two data sets.

 Figure 6 needs a legend, Guo & Yu 2015 is a book chapter and not freely available.

 Your description of how to assign a grid cell ID is a bit over the top in my view and not worth mentioning or maybe shorten in to one sentence and add to the introduction. Geographic Information Systems are around for 40 years now and any GIS works with grid cell IDs, they might just not be in the order of your IDs.

I have read this submission. I believe that I have an appropriate level of expertise to confirm that it is of an acceptable scientific standard.

# Peer review: Referee response for version 1

**DOI:** 10.5256/f1000research.10433.r17421

**Published:** 2016-11-04

**Authors:** Paul West, Peder Engstrom

**Affiliations:** 1 Global Landscapes Initiative, Institute on the Environment, University of Minnesota, Minneapolis, MN, USA; 2 Global Landscape Initiative, Institute on the Environment, University of Minnesota, Minneapolis, MN, USA

CELL5M works toward solving a major problem in the area of food policy decision making; working with the glut of disparate data with differing spatial and temporal resolutions to identify accurate insights relevant to policy makers. Koo and colleagues provide a excellent overview of the CELL5M database. The paper also provides the basic information on the methods used to harmonize the multiple data sets. 

 It appears that the CELL5M team has thought through most of the pitfalls of decision making at this scale. Our main concern in presenting this to policy makers is in abstracting some of the possible inconsistencies in data scale. Here are a few specific comments related to this concern:

  

- Is there some acknowledgement where data has been disaggregated from the national scale, yet presented at a smaller scale? While there is no choice but to use spatially mismatched data in this type of work, it should be made very transparent when the data is not presented at its true scale.
- It should also be transparent where indicators are created from source data at multiple scales. Combining national and sub-national data to create a fine-scale indicator can create a false sense of precision.
- Many of the indicators are likely derived from data sets that either have similar features, creating an uber metric. How do you avoid double counting / weighting some features more than others? 
- Was the harmonization mainly spatial or did you also standardize feature names and units? 

 

 More general comments:

- The abstract could be strengthened. It does not explicitly address this paper until after three long sentences. It’s then very general. A few points to make the abstract more concrete include: 
  harmonized 750+ data sets for feature names, units, and spatial resolution. 
  main themes are: w,x,y,z.
  Provide the type of analysis that is possible and how it can be used (generalize one of the nice examples in the section “ Agricultural development domains”). Be explicit that can integrate social, economic, and biophysical data 
- How did you choose among the many data sets that provide similar information? For example, there are a few sources of data on crop production, yield gaps, and market access. Since different primary data (and methods) were used to create the various data sets, you will get different results when they are integrated here. For you audience, it’s probably better to only have a single data source for each feature, but it would be helpful to be clear on your general criteria for which data are included. 
- The unique cell ID is a great feature for integrating multiple data sets. This also allows for faster, more stable queries and spatial operations using the web mapper or offline.

  

 Although we have not yet used it, the CELL5M data set is a great source for harmonized data for accessing, exploring, and analyzing data for the many uses the authors reference (baseline, setting goals, targeting actions, assessing scenarios, etc.).

We have read this submission. We believe that we have an appropriate level of expertise to confirm that it is of an acceptable scientific standard.
